# Revisiting the Role of TRP, Orai, and ASIC Channels in the Pulmonary Arterial Response to Hypoxia

**DOI:** 10.3389/fphys.2018.00486

**Published:** 2018-05-07

**Authors:** Roberto V. Reyes, Sebastián Castillo-Galán, Ismael Hernandez, Emilio A. Herrera, Germán Ebensperger, Aníbal J. Llanos

**Affiliations:** ^1^Unidad de Fisiología y Fisiopatología Perinatal, Programa de Fisiopatología, Instituto de Ciencias Biomédicas, Facultad de Medicina, Universidad de Chile, Santiago, Chile; ^2^International Center for Andean Studies, Universidad de Chile, Santiago, Chile

**Keywords:** store operated channels, receptor operated channels, hypoxia, hypoxic pulmonary vasoconstriction, pulmonary arterial remodeling, pulmonary hypertension

## Abstract

The pulmonary arteries are exquisitely responsive to oxygen changes. They rapidly and proportionally contract as arterial PO_2_ decrease, and they relax as arterial PO_2_ is re-established. The hypoxic pulmonary vasoconstriction (HPV) is intrinsic since it does not require neural or endocrine factors, as evidenced in isolated vessels. On the other hand, pulmonary arteries also respond to sustained hypoxia with structural and functional remodeling, involving growth of smooth muscle medial layer and later recruitment of adventitial fibroblasts, secreted mitogens from endothelium and changes in the response to vasoconstrictor and vasodilator stimuli. Hypoxic pulmonary arterial vasoconstriction and remodeling are relevant biological responses both under physiological and pathological conditions, to explain matching between ventilation and perfusion, fetal to neonatal transition of pulmonary circulation and pulmonary artery over-constriction and thickening in pulmonary hypertension. Store operated channels (SOC) and receptor operated channels (ROC) are plasma membrane cationic channels that mediate calcium influx in response to depletion of internal calcium stores or receptor activation, respectively. They are involved in both HPV and pathological remodeling since their pharmacological blockade or genetic suppression of several of the Stim, Orai, TRP, or ASIC proteins in SOC or ROC complexes attenuate the calcium increase, the tension development, the pulmonary artery smooth muscle proliferation, and pulmonary arterial hypertension. In this Mini Review, we discussed the evidence obtained in *in vivo* animal models, at the level of isolated organ or cells of pulmonary arteries, and we identified and discussed the questions for future research needed to validate these signaling complexes as targets against pulmonary hypertension.

## Introduction

The pulmonary arteries have distinctive properties all along the individual’s life, compared to systemic arteries. During gestation, the gas exchange is carried out by the placenta, the pulmonary vascular resistance (PVR) is high and the pulmonary blood flow is low, receiving less than 10–20% of the combined fetal cardiac output (CO). In fact, fetal pulmonary arteries have a narrow lumen and a thick medial layer with immature smooth muscle cells, increased synthesis and signaling of vasoconstrictors as well as low oxygen tension (PaO_2_) in the fetal arterial blood, among other factors, which contribute to the pulmonary high resistance, low flow state. At birth, PVR quickly decreases and the pulmonary blood flow increases ∼10 times to accommodate the totality of the CO, allowing the lungs to replace the placenta as gas exchanger at the first minutes of postnatal life (Rudolph, 1979; Suresh and Shimoda, 2016). Increases of PaO_2_ and signaling mechanisms favoring the vasodilation contribute to this fetal to neonatal physiological transition. The pulmonary arteries also undergo a quick and dramatic structural transition at birth: the pulmonary artery smooth muscle cells (PASMC) of the medial layer flattens and their cytoskeleton reorganizes deriving in wall thinning and luminal enlargement. These changes finally allow the establishment of postnatal pulmonary circulation with thin pulmonary arterial wall and low mean pulmonary arterial pressure (mPAP ∼8–20 mmHg at rest for humans) compared to systemic circulation (Peñaloza and Arias-Stella, 2007; Gao and Raj, 2010; Gao and Raj, 2011; Nayr and Lakshminrusimha, 2014). A unique feature of the pulmonary arteries is their intrinsic sensitivity to hypoxia, possessing a vasoconstrictor response to acute hypoxia called “hypoxic pulmonary vasoconstriction” (HPV), which is already present in fetal pulmonary arteries and continues to exist in every life stages. HPV is a rapid and reversible contraction of pulmonary arteries, whose intensity is proportional to the degree of hypoxia and it occurs independently of neural or humoral factors (Sylvester et al., 2012). HPV contributes to the physiologically high PVR state during fetal life, while it allows to optimize the ratio between ventilation and perfusion during the postnatal life (Dunham-Snary et al., 2017; Hussain et al., 2017). Pulmonary arteries also mount a maladaptive response to chronic hypoxia, known as pathological pulmonary arterial remodeling, characterized by the hyperplasic and hypertrophic thickening of medial layer and later thickening of adventitia and intima, with increased extracellular matrix deposition. An example of the later is the exposure to chronic hypoxia during pregnancy, early birth or adult life, resulting in pulmonary hypertension, a condition characterized by elevated pulmonary arterial mPAP and PVR, pathological pulmonary artery remodeling, often complicated with right ventricular hypertrophy and cardiac failure (Herrera et al., 2015; Suresh and Shimoda, 2016).

## Store Operated Channels, Receptor Operated Channels, Structure, Function, and Pharmacology

Cytosolic free Ca^2+^ [(Ca^2+^)i] is pivotal for PASMC contraction, differentiation and proliferation, as well as for synthesis of vasoactive compounds from the pulmonary artery endothelial cells (PAEC). The [Ca^2+^]i may increases through its release from sarcoplasmic reticulum (SR) stores mediated by ryanodine receptors (RyR) or Inositol triphosphate receptors (IP3R), or through calcium influx mediated by voltage operated calcium channels (VOC), receptor operated calcium channels (ROC), or store operated calcium channels (SOC) from plasma membrane (Kuhr et al., 2012).

The SOC is a functional definition for plasma membrane cationic channels that are physiologically activated as calcium stores of SR are gradually depleted by agonists coupled to IP3R or RyR, ensuring a mechanism to refill intracellular Ca^2+^ stores and enabling to maintain long term [Ca^2+^]i signals. The most useful strategy to evaluate SOC function is to measure [Ca^2+^]i in isolated cells pre-loaded with a fluorescent Ca^2+^-sensitive dye like FURA-2AM. The cells are superfused in a Ca^2+^-free medium with nifedipine to block VOC, intracellular calcium stores are passively depleted by inhibition of SR Ca^2+^ pump (SERCA) with thapsigargin or cyclopiazonic acid, and [Ca^2+^]i changes are evaluated after restoration of extracellular Ca^2+^. This Ca^2+^ influx is referred as store operated calcium entry (SOCE). Alternatively, SOCE is also evaluated as the rate of fluorescence quenching by Mn^2+^ which enters the cell after store depletion as Ca^2+^ surrogate and reduces fluorescence through binding the dye (Bird et al., 2008). Concerning their structure, early studies suggested that SOC were formed by pore-forming subunits of the canonical transient receptor potential (TRPC) proteins. However, posterior discoveries showed a complex formed by Orai1 protein and stromal interacting molecule-1 (Stim1) as basic components able to generate store operated calcium influx, raising a controversy about the real molecular identity of SOCs (Earley and Brayden, 2015). This model suggests that SOC are hexamers of Orai proteins (Orai1, 2 or 3) and/or tetramers of TRPC proteins (TRPC1, 3, 4, 5, 6, or 7) that form the pore, and need the interaction mainly with Stim1 protein as calcium sensor of the SR to be activated. Store depletion of SR calcium results in redistribution of Stim1 from a homogeneous pattern in the bulk SR in resting cells, to a localized pattern into regions of SR called *punctae*, close to plasma membrane, allowing its interaction with Orai1 or TRPC proteins and their gating. Stim1-Orai complexes generate the Ca^2+^-release-activated Ca^2+^ currents (I_crac_) associated with oscillations of Ca^2+^ and characterized as a small inwardly rectifying and selective Ca^2+^ currents in electrophysiological studies. These oscillations are necessary to further recruit TRPC subunits, mainly TRPC1, to form Stim-TRPC complexes that generate additional but sometimes less selective Ca^2+^ currents. The selectivity probably depends on the type of TRPC subunits that tetramerize to form the active complex. The recruitment of both currents results in a sustained Ca^2+^ elevation termed the store operated calcium current (I_soc_) (Ambudkar et al., 2017; Putney, 2017). This model is consistent with the ability of different sub-types of Stim, TRPC, and Orai proteins to generate SOCE in pulmonary arteries (Fernandez et al., 2012; Earley and Brayden, 2015; Wang et al., 2017). Moreover, other TRP channels such as transient receptor potential vanilloid-4 (TRPV4) and structurally unrelated Na^+^- and Ca^2+^-permeable cation channels such as acid-sensing ion channels-1 (ASIC1) may also contribute to SOC signaling in PASMC (Goldenberg et al., 2015; Jernigan, 2015). Whether the mechanism linking store depletion and ASIC1 activation to generate SOCE is related with association of ASIC1 with Stim, Orai, TRPC proteins, or other unknown signal has not been elucidated. Next to SOC complexes, TRP proteins also work as ROC. After receptor activation by agonist binding, phospholipase C (PLC) isozymes convert phosphatidylinositol-4,5-bisphosphate (PIP2) into inositoltriphosphate (IP3) and diacylglycerol (DAG), and DAG or its synthetic analog OAG, used to identify receptor operated Ca2+ entry (ROCE) are common activators of all TRPC channels (Dietrich et al., 2005; Fernandez et al., 2012; Storch et al., 2017) except TRPC1 whose role as an ion channel or channel regulator is still a matter of debate (Dietrich et al., 2014).

Inhibitors targeting Stim, Orai, and TRP proteins are increasing in number and potency. Nevertheless, for many of these molecules, their mechanism of action, specificity, and toxicity needs further investigation to allow *in vivo* assays or clinical trials. For instance, two old useful inhibitors are SKF-96365 and 2-APB, are reported to block TRPC3/5, and the Stim/Orai interaction, respectively, at micromolar concentrations, but they also block VOC and IP3R at a similar concentration range (Putney, 2010; Bon and Beech, 2013). Lanthanides, such as La^3+^ or Gd^3+^ strongly inhibit Orai but their use is limited because their water solubility is poor in the presence of proteins and multivalent anions (Bird et al., 2008). Other blockers such as ML-9, BTP2, some GSK-compounds and RO2959 target other molecules in addition to Stim, Orai, or TRP subunits and/or are poorly soluble in physiological solutions (Prakriya and Lewis, 2015; Tian et al., 2016). Some recently characterized inhibitors show improved potency and selectivity: compound 8009-5364 and larixyl acetate block TRPC6 OAG-induced currents (Urban et al., 2012, 2016), AncoA4 blocks Orai channels and prevents its binding with Stim1 (Sadaghiani et al., 2014) while GSK2193874, GSK2220691, and HC067047 block TRPV4 currents (Everaerts et al., 2010; Thorneloe et al., 2012; Balakrishna et al., 2014). These inhibitors are promising tools to study the role of these channels on pulmonary vascular function (**Table 1**). The development of new agents specific for other TRP or Orai isoforms, combining potency and water-solubility should be helpful to study the composition and stoichiometry of native SOC/ROC complexes in pulmonary arteries and to validate them as potential pharmacological targets for pulmonary hypertension treatment.

**Table 1:** Current inhibitors of store operated channels and receptor operated channels.

Inhibitor/type	Observed target or action	Potency	Selectivity	Solubility	Reference
La^3+^, Ga^3+^/Lanthanides	Block pore of Orai1 and Orai3 channels	IC_50_ = 0.2–0.5 μM	Selective at effective concentrations to block Orai	Water soluble, precipitates in the presence of soluble proteins and polyvalent anions	Bird et al., 2008; Prakriya and Lewis, 2015
SKF-96365/phenyle-thyl-imidazole	Block recombinant TRPC3 and TRPC6 currents and Icrac currents	IC_50_ = 0.6–14 μM	Block voltage-gated calcium channels (T-type) and cAMP-gated-chloride channels	Water soluble	Putney, 2010; Bon and Beech, 2013; Tian et al., 2016
2-APB/organoborated	Inteference of Stim1-Orai1 interaction	IC_50_ = 10 μM	Inhibits IP3R, TRPM7 channels, connexins gap junction	Dimethyl sulfoxide (DMSO)	Putney, 2010; Prakriya and Lewis, 2015
ML-9/pyrazol derivative	Inhibits Stim1 translocation	IC_50_ = 10 μM	Inhibits MLCK, PKA and PKC	DMSO	Prakriya and Lewis, 2015; Tian et al., 2016
BTP2/pyrazol derivative	Blocks recombinant TRPC3 and TRPC5 currents and Icrac currents	IC_50_ = 0.1 μM	Activates TRPM4 channels	DMSO, Ethanol	Prakriya and Lewis, 2015; Tian et al., 2016
RO2959	Blocks recombinant Stim1/Orai1, Stim1/Orai2 and Stim1/Orai3 currents	IC_50_ = 25 nM for Orai1 currents and 530 nM for Orai3 currents	Other targets not described	DMSO	Prakriya and Lewis, 2015
GSK5498A, GSK5503A, GSK7975A/pyrazol derivatives	Block recombinant Stim1/Orai1 and Stim1/Orai3 currents	IC_50_ = 1–4 μM	Block TRPV6 channels	DMSO	Prakriya and Lewis, 2015; Tian et al., 2016
GSK2193874/piperidin derivative	Blocks TRPV4 currents and Ca^2+^ entry	IC_50_= 2–40 nM depending on species	Block hERG and CaV1.2 channels with low potency	DMSO	Thorneloe et al., 2012
GSK2220691	Blocks TRPV4 currents	IC_50_= 2.5–10 nM	Selective	0.5% methylcellulose	Balakrishna et al., 2014
HC067047	Blocks TRPV4 currents	IC_50_= 17–133 nM depending on species	Blocks hERG K^+^ channels and TRPM8	DMSO, ethanol	Everaerts et al., 2010
8009-5364/β-carboline derivative	Blocks TRPC6/TRPC3 OAG-induced and agonist-induced Ca^2+^ entry	IC_50_ = 5 μM and 13 μm for OAG-stimulated TRPC6 and TRPC3, respectively.	Blocks TRPA1 channel	DMSO	Urban et al., 2012
Larixyl acetate/larch-derived diterpene	Blocks TRPC6/TRPC3 OAG-induced and agonist-induced Ca^2+^ entry	IC_50_ = 0.58 μM and 6.38 μm for OAG-stimulated TRPC6 and TRPC3, respectively.	Block TRPC7, TRPC4, and TRPC5 with IC_50_ at 2.9–19.3 μM range	Chloroform, ethyl acetate	Urban et al., 2016
AncoA4/tricyclic chromone-fused analog	Blocks Orai1 Ca^2+^ entry and prevents Stim1/Orai1 binding	IC_50_ = 0.88 μM	No other targets described	DMSO	Sadaghiani et al., 2014


*The IC_*50*_ values reported correspond to endogenous Icrac currents or to heterologous expressed TRPC/V, Orai, or Stim currents.*

## The Role of TRP, Stim, Orai, and ASIC Proteins in the Pulmonary Arterial Response to Acute Hypoxia

The SOCE and different TRP, Orai, Stim, and ASIC proteins are robustly expressed in rodent pulmonary arteries and PASMC (Lin et al., 2004; Wang et al., 2006, 2017; Jernigan et al., 2009; Fernandez et al., 2015). Indeed, in PASMC from distal pulmonary arteries the increase of [Ca^2+^]i in response to acute hypoxia, SOCE and the expression of Stim1, TRPC1, 4, and 6 are greater than in proximal pulmonary arteries (Lu et al., 2008). Moreover, evidence from knockdown, knockout, or overexpression experiments show that in rodent PASMC, Stim1 and 2, Orai 1, 2 and 3, TRPC1 and 6, and ASIC1 contribute to SOCE (Lin et al., 2004; Jernigan et al., 2009; Nitta et al., 2014; Fernandez et al., 2015; Wang et al., 2017). Both, hypoxia-induced [Ca^2+^]i increase and HPV responses, are biphasic with a rapid and transient first phase of 5–20 min, followed by a slower and more sustained second phase of more than 180 min (Sommer et al., 2016). The first phase is abolished in TRPC6-/- mice, while knockdown of Stim1 suppresses the second phase (Weissmann et al., 2006; Lu et al., 2009). HPV, SOCE, and ROCE are also attenuated in ASIC1-/- mice compared to ASIC+/+ controls (Nitta et al., 2014). In lung of adult rat, SOC blockade with SKF-96365 inhibits HPV in a concentration-dependent manner (Weigand et al., 2005). The first phase of HPV is also suppressed by TRPC6 blockade with compound 800-5364 and larixyl acetate in mouse isolated lung (Urban et al., 2012, 2016). A significant decrease of HPV is also observed in knockout mice for transient receptor potential vanilloid-4 channel (TRPV4), and there is evidence of “promiscuous” TRPC6/TRPV4 heteromers assembly to generate SOC (Goldenberg et al., 2015). Transcripts of TRPC1, 3, 5, and 6 are detected in PASMC from late gestation ovine fetuses (Resnik et al., 2007) while TRPC1, 3, 4, 5, 6, Orai1, and Stim1 messengers are expressed in lungs from newborn lambs. Moreover the HPV recorded *in vivo* is significantly suppressed through SOC blockade with 2-APB in lambs (Parrau et al., 2013). Taken together, these data clearly show that at least in neonatal sheep and in adult rodents, SOC/ROC are key for contractile response to acute hypoxia, and that at least Stim1, TRPC6, and TRPV4 form part of the molecular complex involved in HPV. Nevertheless, as active Orai and TRPC complexes are hexamers and tetramers, respectively, the possibility of heteromeric association incorporating other Orai or TRPC subtypes to generate Ca^2+^ influx associated to HPV cannot be excluded.

Currently, the mechanism linking hypoxia and SOC/ROC activation is a matter of research. For instance, increase of reactive oxygen species (ROS) during hypoxia is proposed to directly and indirectly activate RyR to deplete SR calcium stores, activate SOC, and increase [Ca^2+^]i and contraction (Sommer et al., 2016; Suresh and Shimoda, 2016). Hypoxia and ischemia/reperfusion provokes DAG accumulation and TRPC6 activation in PASMC and PAEC, respectively, where H_2_O_2_ directly and indirectly mediates this effect (Weissmann et al., 2006, 2012). Interestingly H_2_O_2_ resulting from increased ROS, also promotes the interaction of Stim1, with Orai1 and TRPC1, and upregulates these proteins to mediate SOCE in PASMC (Chen et al., 2017). H_2_O_2_ also promotes Src family kinase-mediated stimulation of TPRV4 in lung microvascular endothelial cells (Suresh et al., 2015), but it is not elucidated if this mechanism also occurs in PASMC. It also remains to be demonstrated if the rate and the potency of the responses evoked by H_2_O_2_ is consistent with tension development observed in HPV. Indeed, in PASMC, [Ca^2+^]i evokes contraction through its binding to calmodulin (CaM) and activation of myosin light chain kinase (MLCK), to phosphorylate the 20 kDa myosin light chain (MLC_20_), and increase the pMLC_20_/MLC_20_ ratio (Ogut and Brozovich, 2008; Kuhr et al., 2012). Despite this obvious link between [Ca^2+^]i and contraction, the relation between pMLC_20_/MLC_20_ ratio and SOC has been demonstrated only for TRPV4 (Goldenberg et al., 2015), while it has not still been demonstrated for other SOC forming subunits. Further, the relation of SOC/ROC with other mechanisms regulating PASMC contraction such as calcium sensitization remains unexplored.

## TRP, Stim, Orai, and ASIC Proteins: Tension Development and Pulmonary Arterial Remodeling in Response to Chronic Hypoxia

The pathological pulmonary arterial remodeling induced by chronic hypoxia is the result of an imbalance between proliferation and apoptosis of PASMC, changes in the differentiation state of PASMC and fibroblasts, and secretion of vasoactive compounds from PAEC (Suresh and Shimoda, 2016).

The PASMC and pulmonary arterial rings from rats exposed to chronic hypoxia have increased basal [Ca^2+^]I and arterial tension, which are reduced by SOC blockade. In contrast, SOC blockade have minimal effects on normoxic controls. Interestingly, protein levels of Orai1 and 2, TRPC1 and 6, Stim1 and 2, ASIC1, and SOCE are greater in pulmonary vasculature of hypoxic animals than control rats (Lin et al., 2004; Wang et al., 2006, 2009, 2017; Jernigan et al., 2012; Hou et al., 2013; He et al., 2018). ASIC1-/- mice show decreased SOCE and attenuated hypoxic pulmonary hypertension compared to ASIC+/+ controls (Nitta et al., 2014). Moreover, chronically hypoxic newborn lambs gestated and born at 3,600 m altitude, have pulmonary hypertension, increased HPV, and upregulated TRPC4 and Stim1 pulmonary transcripts. In these animals, SOC blockade with a single dose of 2-APB evokes a greater attenuation of HPV compared to normoxic controls (Parrau et al., 2013). In another ovine model with partial gestation under chronic hypoxia, the newborn lambs show pulmonary hypertension that persists at sea level and increased TRPC4 and Orai1 expression. Further, an experimental therapy with 2-APB reduces mPAP, PVR, and pathological pulmonary arterial remodeling, both at the level of the medial and adventitial layer in these lambs (Castillo-Galán et al., 2016).

For TRPC1 and 6, as for Orai2, the hypoxic upregulation depends on hypoxia inducible factor-1α (HIF-1α), a cardinal transcription factor involved in the control of oxygen-regulated genes (Wang et al., 2006, 2017). An additional increase of TRPC1 transcription is mediated by nuclear factor of activated T-cells c3 (NFATc3), a calcium-sensitive transcription factor, allowing an amplification of the initial hypoxic induction (Wang et al., 2009). Induction of ASIC1-dependent SOCE by chronic hypoxia is due to increased membrane localization mediated by RhoA activation rather than increased transcription (Herbert et al., 2017). In addition, hypoxia may induce the synthesis and secretion of mitogens from PAEC. These mitogens function as paracrine regulators of PASMC proliferation, and SOC may contribute to both syntheses of these mitogens as to their proliferative signaling. For instance, hypoxia upregulates SOCE and TRPC4 in PAEC. This Ca^2+^ influx increases DNA binding activity of AP-1, a calcium-sensitive transcription factor, to activate transcription of AP-1 responsive genes like ET-1, PDGF, and VEGF among others. This is a mechanism that may contribute to pulmonary artery obliteration observed in pulmonary hypertension (Fantozzi et al., 2003). In turn, mitogens like platelet-derived growth factor (PDGF) and bone morphogenetic protein-4 (BMP4), a secreted ligand of the TGF-β superfamily, bind to receptors on PASMC. In doing so, PDGF and BMP4 further upregulate TRPC1, 4, 6, Orai1, and Stim1 expression, SOCE, and proliferation. PDGF-mediated upregulation of Orai1/Stim1 depends on Akt/mTOR proliferative pathway in both PASMC and pulmonary artery fibroblasts, while BMP4-mediated induction of TRPC1/4/6 depends on p38MAPK-ERK1/2 signaling (Ogawa et al., 2012; Zhang et al., 2014).

Other signaling mechanisms such as extracellular calcium sensing receptor (CaSR) and peroxisome-proliferator activated receptor-γ (PPAR-γ) are also involved in the pathogenesis of pulmonary hypertension and remodeling through SOCE. CaSR are G-coupled protein receptors activated by extracellular Ca^2+^ binding, and they are functionally coupled to TRPC6 activation to promote SOCE and ROCE, among other signaling mechanisms. CaSR are overexpressed in proliferating PASMC from hypoxic rodents or from patients with idiopathic pulmonary hypertension (IPAH), while their pharmacological or genetic suppression reduce proliferation and pulmonary hypertension (Yamamura et al., 2012, 2015; Smith et al., 2016; Tang et al., 2016). PPAR-γ is a member of the nuclear receptor hormone super family, and it is downregulated through TGF-β1 signaling in PASMC, whereas TRPC1 and 6 are upregulated in neonatal and adult rodents with hypoxic pulmonary hypertension. Conversely, stimulation of PPAR-γ reverses pulmonary hypertension and remodeling, and down regulates SOC expression (Gong et al., 2011; Yang et al., 2015; Jiang et al., 2016; Du et al., 2017).

**FIGURE 1:**
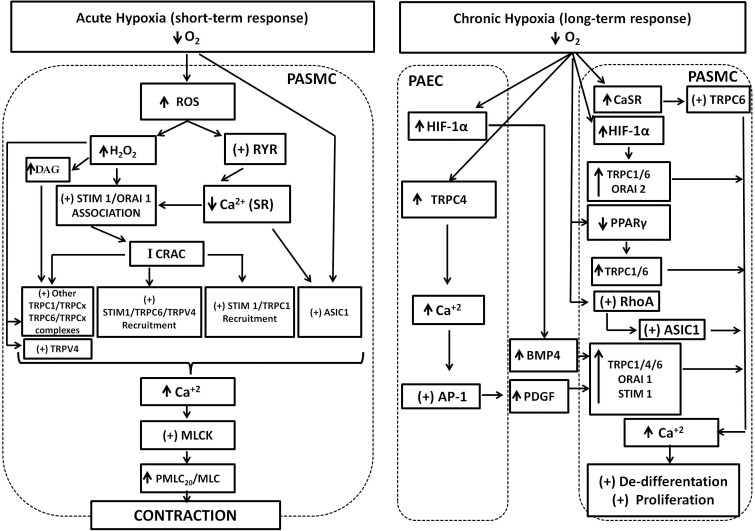
SOC and ROC involvement in the pulmonary response to acute and chronic hypoxia. Acute hypoxia generates increase of ROS and H_2_O_2_. ROS stimulates RyR opening and depletion of SR-Ca^2+^ stores. Both H_2_O_2_ and depletion of SR-Ca^2+^ stores stimulates Stim1/Orai1 association to generate the calcium-release activated calcium current (Icrac), which in turn promotes further interaction of Stim1 with TRPC1, TRPC6, and TRPV4 proteins and recruitment to generate the store operated calcium (Isoc) current. The participation of other TRPC proteins in association with TRPC1 or 6 to generate Isoc cannot be discarded. Increase of DAG cell content promoted by H_2_O_2_ activates TRPC6/TRPCx channels. H_2_O_2_ can also directly activate TRPC6 and TRPV4 channels. Finally, ASIC1 also mediates hypoxic increase of SOC through an unknown mechanism. Increase of SOC and ROC results in the final increase of Ca^2+^, stimulation of the myosin light chain kinase (MLCK), phosphorylation of myosin light chain, and contraction. Chronic hypoxia upregulates hypoxia inducible factor (HIF1) resulting in increased expression the secreted ligand bone morphogenetic protein-4 (BMP-4) from pulmonary artery endothelial cells (PAEC). Hypoxia also upregulates TRPC4 and Ca^2+^ increase, stimulates activator protein1 (AP-1) that in turn upregulates platelet-derived growth factor (PDGF). Secreted PDGF upregulates TRPC1, 4, 6, Orai1, and stim1 in pulmonary artery smooth muscle cells (PASMC). Hypoxia also upregulates the calcium sensing receptor (CaSR) coupled to TRPC6 stimulation and downregulates PPAR-γ in PASMC. Chronic hypoxia also stimulates RhoA protein, which stimulates ASIC1 incorporation to de membrane. The net result is an increase of expression and activity of TRPC1, 4, 6, Orai 1, 2, and ASIC1. The contribution of all these proteins to store and receptor operated calcium entry results in sustained Ca^2+^ increase to promote PASMC proliferation and remodeling.

To proliferate, PASMC undergo a de-differentiation, from a contractile and quiescent phenotype present in functional and healthy pulmonary vessels, to a synthetic and proliferative type, present in later stages of pathological remodeling. TRPC6, Orai2, and Stim2 contribute to the transition from the contractile to the synthetic phenotype (Fernandez et al., 2015). Theoretically, proliferative increase in response to SOCE may be mediated by direct stimulation of calmodulin kinase (CaMK) and p38-MAPK pathway, and activation of Ca^2+^-sensitive transcription factors, such as NFATc3, CREB, and NF-Kβ (Kuhr et al., 2012). NFATc3 has been better studied in relation to PASMC proliferation and remodeling. Dephosphorylation of NFATc3 through Ca^2+^ and calcineurin promotes its nuclear translocation, to activate responsive genes related to PASMC proliferation, apoptosis resistance, and synthesis of contractile proteins, such as α-actin smooth muscle. Chronic hypoxia stimulates NFATc3 nuclear import. Parallel increase of ET-1 synthesis and activation of the RhoA-Rho kinase (RhoA/ROCK) pathway potentiates calcineurin-NFATc3 import (de Frutos et al., 2007, 2011; Ran et al., 2014). Moreover, the anti-proliferative effect of phosphodiesterase-5 (PDE5) inhibition with sildenafil on PASMC occurs with simultaneous inhibition of NFATc3 translocation, decreased SOCE, and TRPC1 downregulation. Additional SOCE decrease evoked by sildenafil may also be explained by PKG-dependent phosphorylation and inhibition of TRPC6 (Wang et al., 2009; Koitabashi et al., 2010). Conversely, the silencing of Stim1 reduces the proliferation of PASMC together with a decrease of NFATc3 translocation (Hou et al., 2013). Decreased NFATc3 nuclear import is also detected after genetic or pharmacological suppression of ASIC1-mediated SOCE (Gonzalez Bosc et al., 2016). Collectively, these data show that Ca^2+^ handling mediated by SOC/ROC stimulate PASMC proliferation through multiple signaling pathways. Several of these transduction pathways need further characterization related to the type of SOC/ROC complexes involved as well as for possible cross talk between them. **Figure 1** depicts the principal links of SOC/ROC with the pulmonary artery response to both acute and chronic hypoxia.

## Conclusion and Perspectives

Identification of Stim, Orai, and TRP proteins as part of the molecular components of SOCE/ROCE, and description of their direct or indirect regulation by oxygen allowed to gain understanding on their contribution to both hypoxic pulmonary vasoconstriction and remodeling. Nevertheless, some challenging developments are still needed in this field to fully understand SOC and ROC function in pulmonary arteries.

Despite Stim1, TRPC1, TRPC6, and TRPV4 are identified part of the SOC and ROC signaling complexes involved in HPV in pulmonary arteries, association of these subunits with other TRPC subtypes to form functional tetramers cannot be excluded. The same observation is valid for Orai complexes. The exact stoichiometry of native SOC and ROC complexes, if different homo/hetero multimer variants Orai, TRP, or ASIC are expressed along the development, and if they are associated to different SOCE and ROCE kinetics, is not elucidated.

Another question relates to the mechanism linking SR store depletion to ASIC1-mediated Ca^2+^ entry in PASMC. It is not known whether this mechanism is related to Stim as Ca^2+^ sensor of the SR, to association of ASIC1 with TRP proteins or to a totally different signal. The possibility to activate ion channels in response to store depletion by mechanisms different to those already discussed here cannot be ruled out.

There is no doubt that continuous chronic hypoxia upregulates Stim, Orai and TRPC proteins, SOCE/ROCE and evokes PASMC proliferation. Nevertheless, if different Stim, Orai, and TRPC proteins are responsive to hypoxia depending on development and duration of the stimulus is an unsolved question. Another form of hypoxia exposure is chronic intermittent hypoxia (CIH), for instance in obstructive sleep apnea (Iturriaga et al., 2014) or intermittent exposition to hypobaria (Herrera et al., 2015). Moreover, rats and mice exposed to CIH develop pulmonary hypertension and pathological remodeling, but the role of Ca2+ influx mediated by Stim, Orai, and ASIC channels on this condition is unexplored (Nisbet et al., 2009; Nara et al., 2015).

SOC and ROC are promising targets against pulmonary hypertension. There is an increasing number of inhibitors targeting Orai, TRP, and ASIC1, but many of them have not been tested in *in vivo* conditions to revert experimental pulmonary hypertension. SOC/ROC have a widespread distribution in the cardiovascular system, and side effects are likely. For instance, a 2-APB treatment decreases mPAP in hypoxic newborn lambs, but CO also diminishes transiently (Castillo-Galán et al., 2016). Therefore, novel SOC inhibitors targeting the pulmonary circulation need to be develop and tested in whole animals with the possibility to simultaneously record the systemic cardiovascular and hemodynamic responses possible to validate them as pharmacological approaches against hypoxic pulmonary hypertension.

## Author Contributions

RR drafted the manuscript. SC-G, IH, EH, GE, and AL edited the manuscript. RR, SC-G, IH, EH, GE, and AL approved the final submission.

## Conflict of Interest Statement

The authors declare that the research was conducted in the absence of any commercial or financial relationships that could be construed as a potential conflict of interest. The handling Editor declared a shared affiliation, though no other collaboration, with one of the authors SC-G.
